# The *Young Lives Matter* study protocol: A case-control study of the determinants of suicide attempts in young people in India

**DOI:** 10.12688/wellcomeopenres.16364.1

**Published:** 2020-11-03

**Authors:** Madhumitha Balaji, Lakshmi Vijayakumar, Michael Phillips, Smita Panse, Manjeet Santre, Soumitra Pathare, Vikram Patel

**Affiliations:** 1 Sangath, H No 451 (168), Bhatkar Waddo, Socorro, Bardez, Porvorim, Goa, 403501, India; 2 Centre for Mental Health Law and Policy, Indian Law Society, Law College Road, Shivajinagar, Pune, 411004, India; 3 Sneha Suicide Prevention Centre, 11, Park View Rd, Bishop Garden, Raja Annamalai Puram, Chennai, 600028, India; 4 Shanghai Mental Health Center, Shanghai Jiaotong University School of Medicine, 3210 Humin Road, Min Hang District, Shanghai, 201108, China; 5 Department of Psychiatry, PCMC's Post Graduate Institute Yashwantrao Chavan Memorial Hospital, Pimpri, Pune, 411018, India; 6 Department of Global Health and Social Medicine, Harvard Medical School, 641 Huntington Avenue, Boston, Massachusetts, 02115, USA

**Keywords:** Suicide attempts, Young people, India, Case-control study, Risk factors, Protective factors, Determinants

## Abstract

Suicide is the second leading cause of death among young people in India. Over 40% of all suicides occur in people between 15 and 29 years of age. Suicide attempts are estimated to be 15 times more common than suicides and substantially increase the risk of subsequent death. However, there has been little systematic study of the determinants for suicide attempts in young people, which makes it difficult to design contextually appropriate and comprehensive suicide prevention strategies for this population. The proposed case-control study seeks to address this knowledge gap by studying a range of risk and protective factors for suicide attempts in young people in India. Field work will be in *Yashwantrao Chavan Memorial Hospital (YCMH) hospital,* in Pimpri-Chinchwad, Pune, India. Cases will be 15-29-year-old individuals admitted to the hospital with self-inflicted non-lethal injuries and poisoning. They will be matched for age and gender with those presenting at the General Medicine outpatient department with other health complaints. In each group, 150 persons will be recruited from YCMH from October 2019 to September 2022 and will undergo a comprehensive semi-structured interview. The primary exposure variable is negative life events over the past 12 months. Secondary exposure variables considered include: demographic characteristics, psychological factors, addictive behaviours, personal resources, adverse experiences over their lifetime, social support, suicidal behaviours in the family and social environment, and exposure to suicide-related information. Data will be analysed using conditional logistic regression. Following completion of the study, workshops will be held with young people, mental health professionals and policy makers to develop a theory of change that will be used to promote suicide prevention. Results will be disseminated via peer-reviewed publications, reports to young people and mental health organisations, and news articles. The study was approved by the Institutional Review Board at Sangath.

## Introduction

Suicide is the second leading cause of death among young people in India. Nearly 60% of all suicides in women and 40% of all suicides in men occur in people between 15 and 29 years of age ^1^. Suicide attempts – non-fatal self-inflicted harm ^2^ - are estimated to be 15 times more common than suicides ^3^, and psychological autopsy studies in India reveal that those who die by suicide are more likely to have made prior suicide attempts than living neighbourhood controls ^4, 5^. The associated psychological impact and substantially increased risk of subsequent death make suicide attempts a significant public health problem.

The Mental Health Care Act of 2017 mandates that the government develop and implement suicide prevention programmes that, among other things, provide treatment and rehabilitation to persons who have attempted suicide. However, there has been limited systematic study of the determinants of suicide attempts in young people in India. The few available investigations either have small sample sizes or are restricted to the assessment of conventional risk factors such as socio-demographic variables, generic life events, and mental illnesses ^6, 7^. There is little information available on risk factors specifically relevant to young people identified in Western studies and other Asian studies, including patterns of thinking and behaviour (e.g., impulsivity, aggression, and substance use); access to information and experiences related to suicide; use of social media or the Internet; and gaming behaviour ^8– 17^. In particular, there is little information on the unique negative life events (NLE) and socio-cultural experiences faced by young people in today’s rapidly changing societies, such as aspirational failures involving romantic relationships, education or employment; living in nuclear families; and greater independence in decision-making about marriage and other matters ^18– 23^. There is also little information in India on protective factors for suicide attempts in young people, such as coping style, religiosity or social support ^24^. We contend that this lack of a holistic understanding of the determinants of suicide attempts in young people makes it difficult to design and implement contextually appropriate and comprehensive prevention strategies that can effectively reduce suicides in this population.

**Figure 1.**
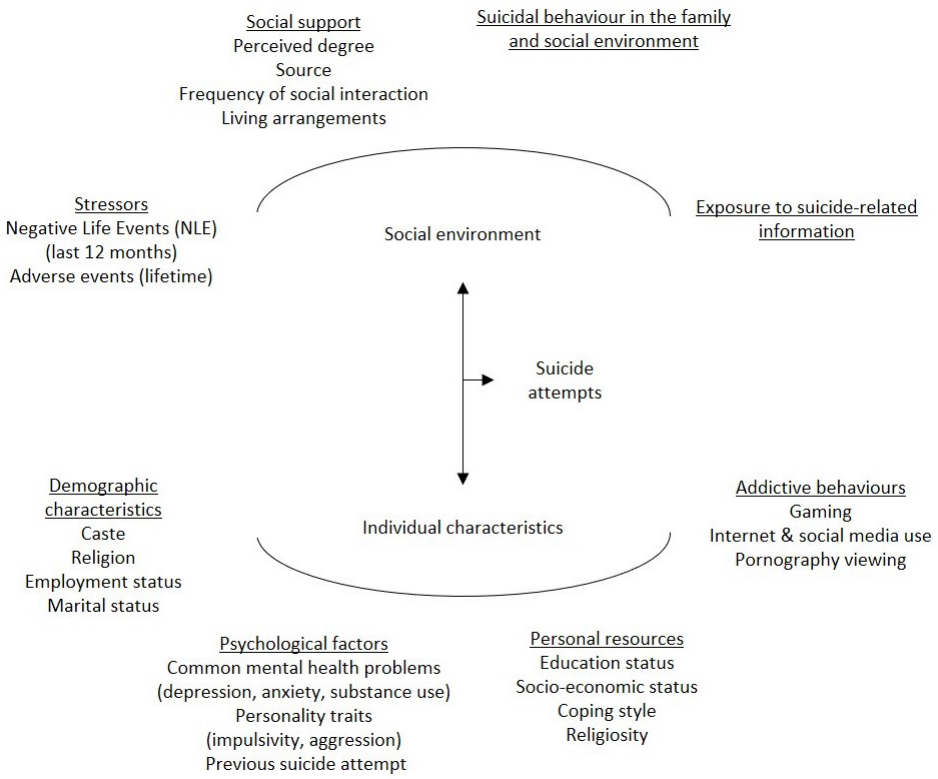
Multi-factor model of suicide attempts (adapted from Phillips *et al*. 1999).

The overall aim of the proposed study is to assess the risk and protective factors for suicide attempts in people 15 to 29 years of age in India. We aim to address current gaps in knowledge about youth suicide in India by studying a constellation of interacting factors that collectively cause suicidal behaviour in young Indians. These factors include individual-level characteristics and experiences in the immediate social environment that were chosen based on an empirically derived multifactorial model of suicide in China ^25^ ( Figure 1). The subsequent goal will be to integrate information garnered from the study to design cohort-specific preventive interventions for Indian youth. The primary exposure of interest is NLE in the last 12 months, as this is a robust predictor of suicidal behaviours in young people ^26^. Our primary hypothesis is that cases are more likely to have a higher number of NLE in the last 12 months than controls. Secondary hypotheses are that cases are more likely to have common mental health problems (e.g., clinically significant depression, anxiety, or substance use problems), suicidal behaviours in the family and social environment, exposure to suicide-related information, and higher Internet and social media use, than healthy controls. An exploratory aim is to examine any differences in determinants by gender, age group and severity of the non-fatal suicidal behaviour.

## Project timelines and phases

The main study commenced in October 2019. Data collection is expected to be completed in 2022 (89 participants have been recruited till date). Data analysis will be completed by 2023.

Prior to the main study, all protocols and procedures were tested for acceptability and feasibility in two pilot phases (July to December 2018, and April to August 2019). Cases were recruited from three hospitals in Phase I – Yashwantrao Chavan Memorial Hospital (YCMH) (large government hospital), DY Patil Medical College Hospital and Research Centre (large private hospital), and Prayag hospital (small private hospital). Only YCMH was involved in Phase II. Two sets of control groups were piloted – neighbourhood controls (phase I), and hospital controls (phase II). There were three groups of hospital controls – in-patients, persons presenting at the Emergency department, and General Medicine outpatients. In total, 62 participants were recruited and interviewed (50 cases and 12 controls).

## Protocol

### Study design

The study uses a case-control design, which is suitable for studying predictors of rare outcomes and for exploring multiple exposures simultaneously ^27^.

### Setting

Recruitment is ongoing at YCMH, in Pimpri-Chinchwad, Pune city, Maharashtra. This 750-bed hospital is owned and operated by the Pimpri-Chinchwad Municipal Corporation established under the Government of Maharashtra. It serves an urban population of about 2 million, nearly 10% of whom live in slum areas. The average literacy rate is 87%. Between 40 and 50 persons with suicide attempts present at the Emergency department of the hospital every month, and approximately 50% of them are treated as outpatients. About 15% come from neighbouring rural areas.

### Study procedures

***Inclusion and exclusion criteria*.** Cases are individuals 15 to 29 years of age who present at the emergency department of YCMH with suicide attempts. A suicide attempt is operationally defined as any non-lethal injury or poisoning that is self-inflicted, and is identified based on entries made in the Medical Legal Case (MLC) records at the Emergency department. These records contain the name, age and gender of all patients whose health concerns are subject to legal investigations, including accidents, injuries, incidents of suspected sexual assault, or violence. Although suicide attempts are no longer a punishable offense according to the Mental Health Care Act 2017, the incident is investigated by hospital authorities and the local Police, in order to confirm that the cause of the attempt is “stress” and there are no political reasons or malpractices involved. Entries from this register are verified with patient records, which contain information regarding the nature of the medical complaint, circumstances surrounding the incident, and the diagnosis made by the treating doctor. In general, accidental injuries and poisoning are entered as ‘accidental’, and suicide attempts are indicated by the method used - ‘OPP’ (organophosphate poisoning), ‘phenyl (household toxin) consumption’, ‘hanging’ and so on. If there is any confusion as to whether an incident is self-inflicted or not (for example, where the circumstances are indicative of self-infliction, but the individual denies this), the decision is made by a mental health professional (SP-YCMH or MB) based on a detailed case narrative.

Inclusion criteria for cases:

(1) Admitted as ‘in-patients’, for stay in a medical ward at the hospital after their suicide attempt

(2) Speak the local languages (English, Marathi or Hindi)

Exclusion criteria for cases:

(1) Have medical or cognitive conditions that impair capacity to participate, for example, persons with speech difficulties, mental retardation, or severe psychosis

(2) Outpatients

(3) ‘Abscond’ (i.e., run away) from the hospital or transferred to another hospital

Outpatients are excluded, because: 1) they frequently leave immediately, often against medical advice, and attempts to contact them afterward are generally unsuccessful because they tend to provide incorrect contact information. Including them would result in high non-response rates, compromising the internal validity of the study. 2) In-patients are the likely recipients of future rehabilitation and suicide prevention efforts in hospital settings, as there is an opportunity to engage with them and their families, and refer them for appropriate psychiatric and community services. Their attempts are also more likely to be medically serious, and their clinical and risk factors more similar to those who die by suicide ^28^.

Controls are persons 15 to 29 years of age who present at the General Medicine Outpatient Department (GM-OPD) of the hospital with health complaints other than suicide attempts. They are matched with cases, 1:1, for age group (15–19, 20–24, and 25–29 years) and gender.

Inclusion criteria for controls:

(1) Outpatients who have medical complaints that are not severe enough to require hospitalization

(2) Speak the local languages (see above)

Exclusion criteria for controls:

(1) Medical or cognitive conditions that impair capacity to participate (see above)

(2) Inpatients (i.e., those who are subsequently admitted to the hospital)

Inpatients are excluded as they typically have serious health complaints, making them more similar to the cases (i.e., ‘over-matching’). Hospital-based rather than community-based controls were chosen ^29– 31^ because they are: 1) from the same study base as the cases, making the research less prone to selection bias; 2) more feasible to recruit than community controls; 3) more likely to be willing to participate than community controls, minimizing non-response bias; and 4) similar to the cases, as they are seeking health care services for a medical condition.

**Figure 2.**
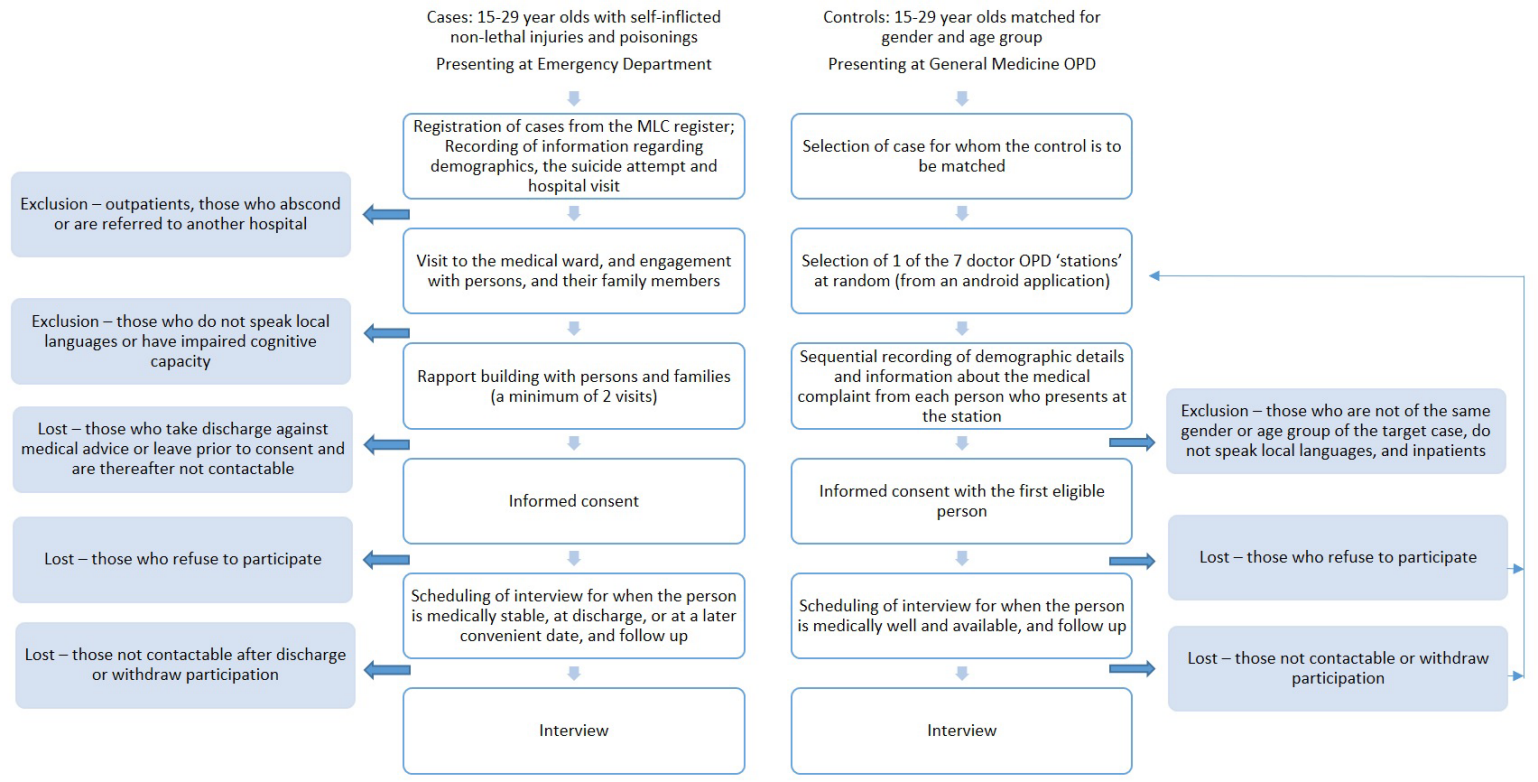
Recruitment process for cases and controls. OPD, Outpatient Department; MLC, Medical Legal Case.

***Recruitment procedures*.** Cases and controls are recruited by Research Assistants (RAs), using the steps described in Figure 2. Cases are recruited every day, all seven days of the week. Controls are recruited during OPD hours (9.30 am to 12.30 pm) on all days except Sundays. There is no recruitment during project or public holidays.

***Informed consent*.** Eligible cases and controls, and accompanying family members are provided with an information leaflet that describes the study in detail, emphasising its importance, potential benefits and risks of participation, procedures for ensuring the confidentiality of collected data, and the freedom to refuse or withdraw without any impact on ongoing medical care. Participants who are willing to participate then sign the consent form and are recruited into the study. If the person is a minor (15–17 years old), the written consent of the legal guardian is obtained, followed by the person’s assent. If the potential case (or his/her guardian) is not literate, informed consent is conducted in the presence of an independent witness, such as a literate family member or someone at the hospital not involved with the study and not treating the person; the person puts their thumbprint on the consent form, and the witness then signs it.

### Sample size estimation

Sample size was estimated using STATA 13 ^32^. To detect a minimum odds ratio (OR) of 2.5, given 80% power, alpha 5%, and baseline exposure of common mental health problems in the controls of 10% ^33^, a sample size of 148 is required in each participant group. This sample size is based on the prevalence of only one of the exposures of interest – common mental health problems. This is presumed to be the exposure with the smallest population prevalence, so this estimated sample size will provide sufficient power to assess the potential significance of other, more common exposures, including NLE.

### Assessment of exposures

All participants are administered a semi-structured interview that was developed based on pilot findings, the survey used in the WHO SUPRE-MISS study ^34^, and consultations with experts on suicide (LV, MP). This interview takes roughly 1.5 hours, and is usually administered in the hospital by RAs who are graduates in clinical psychology or psychiatric social work, and who received six months of training in recruitment and interview procedures. For cases, the date of the attempt is used to calculate the time frame for assessment (12 months, two weeks, etc); for controls, the date of the OPD visit is used. For both cases and controls the interview is completed within two weeks of hospital discharge or outpatient visit. (This relatively short time interval facilitates accurate recall of proximal exposures such as depressive symptoms in the last two weeks.) Most of the data are collected using handheld tablets programmed with the interview but some data, such as narrative descriptions of NLE, are recorded on paper forms. All instruments have been translated and back-translated following WHO guidelines ^35^. Participants who complete the interview receive Rupees 340 (5$USD).

***Primary exposure - NLE in the prior 12 months***

NLE are assessed using a semi-structured questionnaire developed for the study. Participants are asked whether or not 25 NLE have occurred in the last 12 months. These NLE consider nine major life domains: deaths among loved ones, education, employment, relationships, economic, other disappointments, health, legal problems, and traumatic experiences. Both *events* (discrete occurrences) and *episodes* (ongoing/ present stressors) are assessed: examples of events include break-ups of relationships, loss of a job, incurring a large expenditure, or physical abuse; examples of episodes include relationship difficulties, unemployment, taking a loan, or having an alcoholic family member. A detailed narrative of each reported NLE is elicited by the RA who then records standardized information about each NLE: the nature of the NLE, persons involved, and timing (e.g., when it occurred/started; duration). The participant then rates the overall psychological impact of the NLE, using a scale, as: ‘no impact’, ‘mild’, ‘moderate’, ‘severe’, or ‘very severe’.

The 25 NLE were chosen from three sources: the Presumptive Stressful Life Events Scale (PSLES), a life event inventory developed for the Indian context ^36^; previous findings about suicide attempts in India and China ^37, 38^; and pilot findings for the current study. An initial list of NLE was reviewed by experts in adolescent and youth mental health; their suggestions helped select the events included in the final NLE scale used in the study. A comprehensive protocol for administering the new NLE was developed by the PI (MB); it provides strict criteria for determining what constitutes an NLE; how to code NLE; how to differentiate events and episodes; and how to determine the date of occurrence and duration of NLE. The NLE narratives are coded independently by two persons (MB and SP-YCMH), one of whom (SP-YCMH) is blind to the group status (case/control) of respondents. Any discrepancies in coding are resolved through discussion.

***Secondary exposures – Individual***

*Demographic characteristics:* This includes: the *caste* at birth, *religion* (Hinduism/ Islam/ Christianity/ Buddhism/ Sikhism/ Zoroastrianism/ Jainism/ other *)*, *employment status* (student/ homemaker/ employed/ student and employed (for participants who are studying and working at the same time – either of those activities part-time)/ not employed/ other); and *marital status* (single - never married/ with partner – never married/ married – first time/ remarried/ previously married, with partner now/ separated/ divorced/ widowed). These variables and their response options were developed based on national health surveys conducted in India ^39, 40^. Cases and controls are matched on age and gender, so for the purposes of the current study they are not considered potential risk factors.

*Psychological factors:* There are three factors of interest – common mental health problems; personality traits; and previous suicide attempt.

*Common mental health problems* considered include depression, anxiety, and substance use problems.
  Depression and anxiety in the last two weeks: *Depression* and *anxiety* are measured using the Patient Health Questionnaire-9 (PHQ9) and the Generalized Anxiety Disorder-7 (GAD7), respectively ^41, 42^. The PHQ9 includes nine items, one for each of the DSM-IV symptoms of depression, and the GAD7 includes seven items, each measuring one symptom of Generalized Anxiety Disorder. The extent to which these symptoms are present over a two-week period is rated by the participant as 0, 1, 2, or 3 representing 'not at all', 'several days', 'more than half the days' and 'nearly every day', respectively. The total score in PHQ9 ranges from 0 to 27, with 5–9 indicating mild depression, 10–14 indicating moderate depression, and 15–27 indicating severe depression. A cut-off score of 10 indicates clinically significant depression. For GAD7, the total score ranges from 0–21, with a score of 5–9 indicating mild anxiety, 10–14 indicating moderate anxiety, and 15–21 indicating severe generalised anxiety. A cut-off score of 10 indicates clinically significant anxiety. Both instruments have been widely used in India, and are accurate and reliable ^43– 46^.
  Substance use problems in last 12 months. *Alcohol use* is measured using the clinician-administered 10-item Alcohol Use Disorders Identification Test (AUDIT), a screening instrument developed by WHO to detect excessive and harmful drinking ^47^. Items in the AUDIT explore quantity and frequency of drinking (coded in terms of "standard drinks"), alcohol dependence, and problems caused by alcohol use. Each item is scored on a scale of 0 to 4, so the total score ranges from 0 to 40. In India, 8 and above, and 20 and above have been used to indicate probable diagnoses of hazardous drinking and dependence, respectively ^48^. *Tobacco use* is assessed by asking whether the participant has used 1) any form of tobacco in the last 12 months; 2) what types of tobacco had been used; and 3) how frequently it was used (never/ monthly or less/ 2–4 times a month/ 2–3 times a week/ 4 or more times a week).

Symptom inventories were chosen over diagnostic measures, as these yield both continuous and categorical data, take less time to complete, engage the participants better, and are easier to use by RAs who do not have extensive clinical experience.

*Personality traits over the last 12 months*: The two personality traits most frequently associated with suicide attempts ^49, 50^ are assessed:
*Impulsivity* is assessed using the brief Barratt Impulsiveness Scale (BIS-Brief) ^51^, an instrument suitable for both adolescents and adults. It includes eight items from the original 30-item BIS, measured on a 4-point scale from 1 (rarely/never) to 4 (almost always/always).
*Aggression* is assessed using the Buss Perry Aggression Questionnaire, a 29-item inventory that assesses four aggressive behaviours: anger, physical aggression, verbal aggression, and hostility ^52^. Each item measures the extent to which the participant agrees or disagrees with the statement on a scale of 1 ('extremely uncharacteristic of me') to 5 ('extremely characteristic of me'). Each of the four subscales is scored by summing the items. The overall score is the sum of the subscales. Higher scores indicate higher levels of impulsivity and aggression.
  Internal consistency scores (Cronbach alpha) for BIS-Brief and Buss Perry in the pilot were moderate to high; 0.64 and 0.82, respectively.

*Previous suicide attempt over lifetime:* Whether the participant has ever made a suicide attempt (for cases, this would be any *prior* to the index attempt).

*Personal resources:* These are within-person capacities, resources or coping mechanisms that can act as protective factors, and include:

*Education status at time of interview:* The highest successfully completed education level of the participant. The response categories are: no formal education, less than primary, primary, middle school, high school, under-graduate (bachelor’s degree), post-graduate, other.
*Socio-economic status at time of interview*: This is measured using a proxy indicator that is a predictor of health outcomes in India – the highest completed education level of the participant’s mother ^53^.
*Coping style employed over the last 12 months*: This is assessed using the Brief COPE scale ^54^, which considers 14 strategies: self-distraction, active coping, denial, substance use, use of emotional support, use of instrumental support, behavioural disengagement, venting, positive reframing, planning, humour, acceptance, religion, and self-blame. There are two questions per strategy, 28 items in total. The participant rates the extent to which he/she uses a coping strategy on a scale of 1 to 4 ('I haven't been doing this at all' to 'I have been doing this a lot'). Higher scores for each strategy are indicative of greater use of that strategy. Coping strategies can be classified as adaptive or maladaptive ^55^. The scale was found to be reliable and valid in India ^48^, and the pilot and consultations with experts showed good face validity.
*Religiosity over the last 12 months*: The extent to which the participant prays or meditates, and finds comfort in his/her religion and religious beliefs; behaviours that may protect a person against suicide attempts through reduced substance use and better coping mechanisms ^56, 57^. There are two questions in Brief COPE – one for each of the above - and each question measures the extent of these behaviours on a scale of 1–4. The categorical responses to these questions as well as the total scores will be analysed.

*Addictive behaviours:* These include the following behaviours that have recently been associated with suicide attempts in young people:

*Gaming over the last three months*: How much time the participant spends playing video or computer games on a typical day – not at all, less than 30 mins, 30–60 mins, 1–2 hours, 2–3 hours, and so on.
*Internet and social media use over the last three months*: Time spent on the Internet and social media for purposes other than work on a typical day (response categories same as above).
*Pornography viewing over the last three months*: Monthly frequency listening to, viewing or reading any content related to sexual activity in order to experience sexual stimulation - never, monthly or less, 2–4 times a month, 2–3 times a week, 4 or more times a week.

***Secondary exposures – Social***

*Adverse events during lifetime*: This includes experience of *parental death*, *parental divorce/ separation* or *forced marriage* (i.e., whether the participant had to get married against his/her wishes). If these events occurred, the age at which they occurred is recorded; it they occurred in the last 12 months, they are also coded under NLE.

*Social support over the last 12 months*: This is measured as:

*Perceived degree of social support*: The extent to which the participant felt supported by persons in the environment, as measured by the frequency of using emotional and instrumental support (Brief COPE subscales), and the total scores on these items.
*Source of social support*: Who provided this support (significant other, family members, friends, etc.). Multiple sources are indicative of greater support.
*Frequency of social interaction*: How often in a month the participant spent time with someone who he/she does not live with (other than at school or work)- never, monthly or less, 2–4 times a month, 2-3 times a week, 4 or more times a week.
*Living arrangements*: Who the participant lived with for a majority of the time in the last 12 months - alone, friends/roommates, hostel, nuclear family, joint family, other. Living with others, or in joint families are indicators of greater social support.

*Suicidal behaviour in the family and social environment at lifetime:* Whether any of the participant’s blood relatives or other persons in the social environment (other family members, friends, neighbours etc.) had ever attempted or died by suicide. The timing of the most recent suicide, and suicide attempt is coded as last day, last week, last month, last three months, last 12 months, or over 12 months ago.

*Exposure to suicide-related information in the prior month:* Whether the participant accessed or was exposed to any information on suicide and, if yes, what this information was (reports of suicide cases, ways/ means of attempting suicide, where to get lethal substances, what lethal dosages are, etc.) and where the information was accessed (print or internet media reports, YouTube/ other videos, books, social media posts, etc).

### Other data

The following additional data are collected from cases –

*Information about the suicide attempt:* the method of the attempt, where it took place, the time, accessibility of means used, etc.

*Suicide intent:* questions from the WHO-SUPRE MISS study, exploring the extent to which the participant was isolated, timed the intervention to avoid or receive help, planned the attempt, took precautions to avoid discovery, sought help, made arrangements for death, communicated suicidal intent, and left a suicide note.

*Expected outcome:* what they hoped for or expected would happen if they attempted suicide- death, temporary relief, change in others' behaviour, do not know/ did not think about it, other.

*Reason for the attempt:* what they believe lead to the attempt (recorded as verbatim narratives).

*Posting of suicide content on social media in the prior month:* if and what they posted relating to their distress or suicidal intent on social media, where the content was posted, and when the last posting was.

### Defining a serious suicide attempt (SSA)

An SSA is operationally defined as an attempt that meets three criteria – *lethal method, medically serious,* and *high suicide intent* ^58^. *Lethal methods* are those means considered to have high potential for lethality in India including organophosphate (pesticide) poisoning, hanging, and self-immolation ^59, 60^. A *medically serious* attempt is one that requires surgery, or treatment in a specialised unit such as the Intensive Care Unit. *High suicide intent* is an attempt that meets a cut off score of 7 or more on the suicide intent questions.

### Data analysis

Quantitative data will be analysed using SPSS 25 ^61^ and STATA 15 ^62^. Descriptive analysis will be conducted for cases and controls. Bivariate analysis using t tests, and chi-square tests or OR (with 95% confidence interval) will be conducted for numerical and categorical variables respectively. Conditional logistic regression will be used to determine the adjusted OR of statistically significant risk and protective factors. The analysis will be done for the all participants, and also be stratified by age (15–19, 20–24, 25–29 years) and gender (male, female). Any differences in OR for SSA will be examined. Since this is a relatively small dataset, no median or mean values will be imputed for missing data during the analysis. The bivariate analysis will be presented as it is. For multivariate analysis, the pattern of missing values will be examined and decisions will be made as to what variables can be included.

Qualitative data will be analysed using thematic analysis ^63^. Content analysis may also be conducted; this involves examining and quantifying the presence of certain words or concepts within the data ^64^.

### Ethics

The study has been approved by the Institutional Review Board of Sangath (IRB registration number - ECR/235/indt/GA/2015; project registration number – MB_2017_28 (19.08.2017)).

Informed consent procedures described earlier are in accordance with guidelines developed by the Indian Council for Medical Research ^65^. Efforts are made to prevent further suicide attempts in cases, by following recommended guidelines ^66^. The RAs first assess the risk for suicide by examining interview findings regarding recent losses or stressful life events and depressive symptoms, and making additional enquiries about current suicidal ideation or plans, and means available. They then do the following to prevent another attempt: advise participants to use adaptive ways of coping or strategies that have worked for them in the past; recommend that they restrict access to means as long as suicide ideation is present; request that they have in mind specific family members and friends who they can reach out to in times of distress; encourage them to call a suicide help-line in Pune (‘Connecting'); and facilitate a consultation with a psychiatrist in YCMH. They also mobilise support from the family by advising them to remove access to means, closely monitor the participant and not leave him/her alone; and bring him/her for a follow up psychiatric consultation.

### Dissemination

Workshops will be held with young people, mental health experts and policy makers, to identify a "theory of change" (TOC) for suicide prevention ^67^. TOC is a scientific tool that identifies how a series of specific initiatives can achieve a long-term goal by first clearly defining this goal and then working backwards to identify the conditions that are required for this to happen. Participants will be asked to develop a TOC for preventing suicide taking into consideration the risk and protective factors identified through the study. Findings from the study, and from the subsequent workshop will be published in peer reviewed journals. In line with funding policies, data will be made available in Europe PMC soon after publication. Published reports, media articles, and recommended guidelines for suicide prevention will be disseminated to young people, mental health NGOs, and the general public.

## Conclusion

There are three limitations. The first is that the generalisability of findings is limited to young adults who 1) access government hospitals in India (i.e., typically those from lower or middle income strata) 2) come from predominantly urban areas, and 3) tend to have more medically serious attempts (i.e., in-patients and not outpatients). The second is that the study does not consider uncommon mental health conditions such as schizophrenia, because of the challenges involved in conducting a formal diagnostic examination and because of the low prevalence of these conditions. The study also does not consider distal risk factors such as childhood sexual abuse or neglect; this was partly due to the high risk for recall bias associated with reporting such memories ^68^. While assessing the impact of the primary exposure (NLE), the overall impact is considered, not impact at the time of the attempt, and hence acute stress scores cannot be calculated. The third limitation is the potential recall bias and under-reporting of sensitive data by both cases and controls. However, steps have been taken to minimise this possibility ( Table 1).

**Table 1.** Strategies to minimise bias.

Type of bias	Strategies to minimise bias
Selection bias	Cases and controls are selected from the same hospital population. The ‘station’ from which a control is selected on a given day is determined at random. The first eligible person in the queue is selected to participate. Only if this person refuses or is unavailable for two weeks, the procedure is repeated. This ensures that controls are selected systematically and not based on convenience or availability.
Recall bias	The control group is also ‘sick’ but with a different health condition. The time frame for the assessment of the primary exposure variable (NLE) is the last 12 months only. The time frame for the assessment of most of the secondary exposures is also within the last 12 months. Assessment at lifetime is mainly limited to life experiences that are ‘objectively’ measurable (and not prone to differential recall) like parental death or divorce. Exposures that are the ‘subjective’ and, thus, prone to differential recall (mood symptoms, exposure to suicide-related information, use of social media etc) are assessed over shorter time periods: past two weeks/ three months. Most exposures are assessed by the RAs – i.e., are interviewer-rated rather than participant-rated. A comprehensive and standardised protocol guides this rating; for example, information regarding timing of NLE is elicited and coded using a set of standard, pre-determined criteria, applied uniformly across all participants. Where exposures are rated by the participant, visual aids are provided to facilitate understanding, for example, while rating the PHQ9, participants are provided with a card having response options that are green in colour; this colour becomes darker in shade with increase in the frequency of symptoms (‘not at all’ is light green, ‘almost every day’ is dark green). Interviews are conducted in the same setting for both participant groups (hospital). Interviews are completed within two weeks of discharge/ GM-OPD visit, to facilitate accurate recall. Rapport is built with participants in both groups before the interview, to make participants comfortable with reporting sensitive information.
Interviewer bias	The interview is uniformly administered in the same way for cases and controls (the wording of questions, order, etc). The coding of responses is guided by a comprehensive and standardised protocol that is applied uniformly across all participants. NLE narratives are coded by two persons, one of whom is blind to the case-control status. Standardised scales and ‘objective’ indicators are used as far as possible. Interview quality is monitored through weekly supervision sessions.

NLE, negative life event; RA, Research Assistant; PHQ9, Patient Health Questionnaire-9; GM-OPD, General Medicine Outpatient Department.

To the best our knowledge, this is the first study in India that explores the determinants for suicide attempts in 15–29 year-old individuals, an important public health concern regarding which there is little data. A key strength is its comprehensive and standardized assessment of both risk and ** protective factors, including individual characteristics and social environment variables. Moreover, the study considers aspects previously not reported in India, including the sociocultural experiences of young people. Results of the study can aid the development of national suicide prevention programmes in India, an important agenda specified in India’s Mental Health Act 2017.

## Data availability

No data are associated with this article.
