# Supplementary material for: The Young Lives Matter study protocol: A case-control study of the determinants of suicide attempts in young people in India
Source: Wellcome Open Res. 2020 Nov 3;5:262. [Version 1] doi: 10.12688/wellcomeopenres.16364.1 (PMC8042515; doi:10.12688/wellcomeopenres.16364.1)
Supplement: Peer review history

# Peer review: Reviewer response for version 1

**DOI:** 10.21956/wellcomeopenres.17998.r43136

**Published:** 2021-04-12

**Authors:** Philippe Mortier

**Affiliations:** 1 Health Services Research Unit, IMIM-Institut Hospital del Mar d'Investigacions Mèdiques, Barcelona, Spain

This is an excellently written, well-designed, and highly interesting study protocol of a matched case-control study (150 cases; 150 controls) on suicide attempts presenting at the emergency department. An important strength of the study is the fact that semi-structured interviews are conducted with the cases & controls, which is superior over reliance on medical record data, and will highly benefit data quality. I congratulate the authors on their effort, and wish them all the best with their study in the years to come. Below I leave some comments (rather reflections), that could perhaps lead to some minor revisions it the authors see it fit. Thank you for the opportunity to review this study protocol.

 Introduction: the justification for focussing on suicide attempts (at the ED) as the outcome consists of preventing suicides; however, many people with suicide attempts never complete suicide, many completed suicides never contact healthcare, and suicide attempts as such also have other short- and long-term negative consequences, apart from suicide. It may be interesting to add this to the introduction for a more balanced overview of the importance of tackling suicide attempts on the societal level.

 Introduction: "There is little information available on risk factors specifically relevant to young people identified in Western studies". I believe this is not entirely true (I agree with the authors that this may be true for India/Asia). See "Franklin JC, Ribeiro JD, Fox KR, *et al.* (2017) ^1^ for a detailed analysis of risk factors studies in suicide research over the past decades. The problem is that many risk factors are studied over and over again, others are understudied, that there is a lack of consensus/evidence for a unified theoretical framework on the etiology of suicidality, and that there is a lack of research that integrate many risk factors into final exploratory or hypothesis-testing models.

 Protocol: the case definition: how are suicide attempts distinguished from non-suicidal self-injurious behavior at the emergency department? Is suicidal intent assessed? The authors assess suicidal intent in the semi-structured interview; however, what if suicidal intent was absent? Are participants consequently excluded as cases? 

 Protocol: cases are inpatients, while controls consist of outpatients. Why? Would it not be better in terms of comparability to recruit controls from inpatients without suicide attempts? The authors state: "Inpatients are excluded as they typically have serious health complaints, making them more similar to the cases (i.e., ‘over-matching’)." Perhaps the term "over-matching" could be clarified further.

Is the study design appropriate for the research question?

Yes

Is the rationale for, and objectives of, the study clearly described?

Yes

Are sufficient details of the methods provided to allow replication by others?

Yes

Are the datasets clearly presented in a useable and accessible format?

Not applicable

Reviewer Expertise:

Psychiatric epidemiological research; suicide research.

I confirm that I have read this submission and believe that I have an appropriate level of expertise to confirm that it is of an acceptable scientific standard.

## References

[1] : Risk factors for suicidal thoughts and behaviors: A meta-analysis of 50 years of research. *Psychol Bull*.143(2) : 10.1037/bul0000084 187-232 10.1037/bul0000084 27841450

# Peer review: Reviewer response for version 1

**DOI:** 10.21956/wellcomeopenres.17998.r41354

**Published:** 2020-11-17

**Authors:** Paul Moran

**Affiliations:** 1 Centre for Academic Mental Health, Department of Population Health Sciences, University of Bristol, Bristol, UK

I enjoyed reading this paper describing the protocol for this hospital-based case control study of the determinants for suicide attempts among 15–29 year-old individuals. The study is likely to yield new important data on suicidal behaviour in young people in India. 

 Key strengths of the design are the selection of cases and controls from what one assumes is the same ‘at risk’ population, the use of trained raters and standardised assessments and the fact that they are capturing a wide range of individual, social and environmental variables, including contemporary behaviours that have been linked to suicidal behaviour in young people in other populations. I really liked the table outlining the precautions that the team are adopting to guard against bias, which is inevitable in any study design. 

 I have a few minor quibbles that I have listed below:

1. The selection of controls in a hospital-based case-control study is never simple. In this instance, the authors have decided to use outpatient-based controls and justified this on the grounds that inpatient controls would have led to over-matching. Equally, by comparing the exposure status of inpatients admitted following a suicide attempt, with outpatients who presumably have a wide range of diseases (some of which might be linked to negative life events), will also introduce selection bias. On this note, it would be useful to know more about the range of problems typically seen in the outpatient population. This is important in terms of understanding whether these might also be associated with exposure to (or recall of) negative life events, which would bias the effect estimate.
2. Its probably unfeasible for the research team to achieve blind ratings of exposure status because the nature of setting where the interview takes place (i.e. ward or outpatients) would immediately reveal whether someone is a case or a control. However, it would be important to name the lack of blind ratings of exposure status as a limitation.
3. The authors propose to measure a wide range of exposures, including selected aspects of personality – especially impulsivity, which we know is an important vulnerability factor for suicide attempts. However, I would have liked to have seen the inclusion of other key vulnerability and motivational factors – particularly social perfectionism and sensitivity to defeat and entrapment.
4. On a similar note, I would like to have seen clearer justification for their choice of the suicide model underpinning their assessment battery. Other models notably the Integrated Motivational-Volitional Model, may have greater empirical support and would be relevant to this study.

Is the study design appropriate for the research question?

Yes

Is the rationale for, and objectives of, the study clearly described?

Yes

Are sufficient details of the methods provided to allow replication by others?

Yes

Are the datasets clearly presented in a useable and accessible format?

Not applicable

Reviewer Expertise:

Self-harm research.

I confirm that I have read this submission and believe that I have an appropriate level of expertise to confirm that it is of an acceptable scientific standard.
